# Vaccination of infants aged 0 to 11 months at the Yaounde Gynaeco-obstetric and pediatric hospital in Cameroon: how complete and how timely?

**DOI:** 10.1186/s12887-017-0954-1

**Published:** 2017-12-19

**Authors:** Andreas Chiabi, Félicitée D. Nguefack, Florine Njapndounke, Marie Kobela, Kelly Kenfack, Séraphin Nguefack, Evelyn Mah, Georges Nguefack-Tsague, Fru Angwafo

**Affiliations:** 1 Yaounde Gynaeco-Obstetric and Pediatric Hospital, Yaounde, Cameroon; 2 Faculty of Medicine and Biomedical Sciences, University of Yaounde I, Yaounde, Cameroon; 3 Institut Supérieur des Sciences de la Santé, Université des Montagnes, Bangangte, Cameroon

**Keywords:** Immunization timeliness, Immunization completeness, Expanded programme of immunization

## Abstract

**Background:**

Vaccination is a major, but simple and cost effective public health intervention in the prevention of infectious diseases, especially in children. Nowadays, many children still miss scheduled vaccines in the Extended Program of Immunization (EPI) or are being vaccinated after the recommended ages.This study was aimed at assessing vaccination completeness and timeliness in children aged 0 to 11 months attending the vaccination clinic of the Yaounde Gynaeco-Obstetric and Pediatric Hospital.

**Methods:**

This was an observational cross-sectional study over a period of 3 months (1st February to 30th April 2016). 400 mothers were interviewed and their children’s vaccination booklets analyzed. Information on the children and the parents was collected using a pretested questionnaire. Data analysis was done using SPSS version 20 software. Bivariate and multivariate analysis with logistic regression was done to assess the determinants of completeness and timeliness.

**Results:**

A total of 400 mother-infant pairs were sampled. The vaccination completeness rate was 96.3%. This rate varied between 99.50% for BCG and 94.36% for IPV. Most of the children were born at the Yaounde Gynaeco-Obstetric and Pediatric hospital where they were regularly receiving their vaccines. The proportion of correctly vaccinated infants was 73.3%. The most differed vaccines were BCG, PCV13 and IPV. Factors influencing immunization completeness were the father’s profession and the mother’s level of education.

**Conclusions:**

Despite the high immunization coverage, some children did not complete their EPI vaccines and many of them took at least one vaccine after the recommended age.

**Electronic supplementary material:**

The online version of this article (10.1186/s12887-017-0954-1) contains supplementary material, which is available to authorized users.

## Background

Vaccination is considered as one of the biggest achievements of the twentieth century and as one of the most cost effective measures in the prevention of childhood diseases [1]. In 1974, the World Health Organization (WHO) launched a worldwide vaccination program known as the Expanded Program of Immunization (EPI), which has been considered one of the major public health interventions aimed at reducing infant morbidity and mortality [2]. During the launching of the EPI in 1976, only about 5% of infants throughout the world were protected against six diseases (diphteria, measles, pertussis, poliomyelitis, tetanus, and tuberculosis). By 2013, the number of protected infants was more than 80% in many countries. It is estimated that vaccination helps to prevent 2 to 3 million infant deaths each year [3].

The Expanded Program of Immunization started in Cameroon in 1976 as a pilot project and targeted infants from 0 to 11 months. Initially it targeted 6 diseases (diphtheria, measles, pertussis, poliomyelitis, tetanus, and tuberculosis), and other vaccines were gradually introduced; the last to be introduced in the EPI was IPV in 2015. Presently, it has vaccines against the following diseases: tuberculosis, diphteria, tetanus, poliomyelitis, pertussis, viral hepatitis B, type b Hemophilus influenza infections, pneumococcal infections, diarrhoea caused by rotavirus, measles, yellow fever, and rubeola. An infant is completely immunized when he or she has received all the vaccines in the EPI. Ensuring that all the doses are not only administered, but given at the appropriate ages, is of crucial importance in ensuring the efficacy of the vaccine in disease prevention [4]. An infant is correctly vaccinated when he or she has received all the vaccines at the recommended ages. Many infants still do not complete their vaccination schedules or are vaccinated after the recommended ages [5, 6].

Given the importance of vaccination in reducing morbidity and mortality in children, we decided to assess the completeness and timeliness of immunization and its determinants at the Yaounde Gyneco-Obstetric and Pediatric hospital, which is a tertiary mother and child hospital in Cameroon. This will ultimately improve the vaccine coverage and reduce obstacles which might hinder effective implementation.

## Methods

A cross-sectional analytical study was conducted; over a period of 3 months (1st February to 30th April 2016) in the vaccination unit of the Yaounde Gyneco-Obstetric and Pediatric Hospital (YGOPH), which is a mother and child referral hospital in Yaounde, the capital city of Cameroon. All mothers of infants aged 0 to 11 months coming for routine EPI were enrolled in the study.

Pre-tested questionnaires were filled for all mother-infant pairs at the vaccination unit, after obtaining consent from the mothers or caretakers of the infants (see Additional file 1). Information collected on the infants included age, sex, place of birth, place of first vaccination, the usual vaccination site, vaccines received, and date of vaccination for each antigen received.

Information concerning the parents included: age, level of education, profession, marital status, religion, region of origin, distance from the house to the vaccination unit, satisfaction from vaccination unit as expressed by the mothers or caretakers. The cut offs of 30 years for the mothers’ age and a distance of 5 km, was used in our analysis; same cut offs were used by Hu et al. [6]. The mothers or the caretakers of the infants were first interviewed and then the vaccination booklets of the infants they came with examined (to minimize recall bias); to verify the vaccines received and the dates they were administered.

The sample size (N) was determined using the formula: $\frac{{\mathrm{z}}^2\mathrm{p}\;\left(1-\mathrm{p}\right)}{{\mathrm{d}}^2}$

where z is the significance threshold;1.96 for a 95% confidence level, d is the error margin; 5%, and p; 64.3%, is the prevalence of vaccine completeness from the study of Ba Pouth et al. [5] in the Djoungolo health district in 2012.

## Definition of variables

The dependent variables were the immunization completeness, and the antigen specific immunization coverage of children aged 0 to 11 months.

An infant was considered as being completely vaccinated if he/she had received all of the doses of the following vaccines: BCG, OPV_0_, DTP-HepB_1_-Hib1, OPV_1_, Rota_1_, Pneumo13_1_, DTP-HepB_1_-Hib_2_, OPV_2_, Rota_2_, Pneumo13_2_, DTC-HepB_1_-Hib_3_, OPV_3_, Pneumo13_3_, Measles, Yellow fever and Rubeola vaccines according to the EPI schedule.

The immunization coverage per antigen was defined by the ratio of infants that received the antigen divided by the total number of infants sampled.

Immunization timeliness was defined as being vaccinated at the recommended ages. A period of 2 weeks was considered above which the vaccine was considered as delayed. Any child with delayed administration of one or more antigens was considered not timely vaccinated.

The independent variables were the different socio-demographic characteristics of our sample population. The outcomes were immunization completeness and timeliness.

### Data analysis

Data analysis was done using SPSS version 20.0 for windows. The data input control permitted the minimization of errors. The analysis of factors associated to vaccination completeness was done using the ‘backwards’ model of multivariate logistic regression. Logistic regression was first done to obtain the crude odds ratio for each of these factors with their 95% confidence intervals and their *P*-values. Thereafter the variables with a *p*-value <0.2 were all entered in a model of multivariate logistic regression to control the confounding factors and determine which characteristics were independent predictors of the immunization completeness of the child. A p-value <0.05 and an adjusted odds ratio (AOR) with its 95% confidence interval not containing 1.00 was considered significant.

### Ethical considerations

Prior to carrying out this study, administrative authorization and ethical clearance was obtained from the Yaounde Gynaeco-Obstetric and Pediatric hospital and the Faculty of Medicine and Biomedical Sciences of the University of Yaounde I respectively. A written consent form was signed by each mother or caretaker who accepted to be enrolled and participate in the study, and for those who could not read and write verbal consent was sought after receiving information on the study. Participants in the study were informed on any missed vaccine and any other information concerning the child’s vaccinations. All infants with vaccinations not up-to -date were vaccinated as recommended.

## Results

### Socio-demographic characteristics of the study population.

Overall, there were 415 mothers eligible for the study, and 15 were excluded (10 did not consent to participate and 5 did not have vaccination booklets). A total of 400 mother-infant pairs were sampled, of which 203 (51%) were females and 197 (49%) males; giving a sex ratio of 0.97. The median age for the infants was 98 days (range 1 day to 266 days). Most mothers (56.5%) were less than 30 years, 61.3% had secondary education, 79% were married and 50.3% lived at more than 5 km from the vaccination site (see Table 1). Almost all the fathers (94.8%) had at least secondary school education and 38.8% worked in the informal sector (see Table 2).

**Table 1:** Vaccination schedule for children aged 0–11 months in Cameroon [20]

Contacts	Age	Vaccine	Route of administration	Preventable diseases
1st contact	At birth	BCG	Intradermal	Tuberculosis
		OPV 0	Oral	Poliomyelitis
2nd contact	6 weeks	DTP-HepB-Hib 1	Intramuscular	Diphteria, Tetanus, Pertussis,Infection due to Haemophilus Influenzae type b, Hepatitis B
		OPV 1	Oral	Poliomyelitis
		Pneumo 13–1 (PCV)	Intramuscular	Pneumococcal infections
		ROTA 1	Oral	Rotavirus Diarrhoea
3rd contact	10 weeks	DTP-HepB-Hib 2	Intramuscular	Diphteria, Tetanus, Pertussis,Infection due to Haemophilus Influenzae type b, Hepatitis B
		OPV 2	Oral	Poliomyelitis
		Pneumo 13–2 ROTA 2	Intramuscular Oral	Pneumococcal infections Rotavirus Diarrhoea
4th contact	14 weeks	DTP-HepB-Hib 3	Intramuscular	Diphteria, Tetanus, Pertussis,Infection due to Haemophilus Influenzae type b, Hepatitis B
		OPV 3 IPV	Oral Intramuscular	Poliomyelitis
		Pneumo 13–3	Intramuscular	Pneumococcal infections
5th contact	6 to 11 months	Vit A	Oral	
	At 9 months	MR	Subcutaneous	Measles, Rubella
		YF	Subcutaneous	Yellow fever

**Table 2:** Socio-demographic characteristics of the parents

	Variables	Number	Percentage (%)
Mother’s age (years)	< 30	226	56.5
	≥ 30	174	43.5
Mother’s level of education	Illiterate	4	1.0
	Primary	32	8.0
	Secondary	205	61.3
	Higher	159	39.8
Mother’s profession	Private	50	12.5
	Public servant	59	14.8
	Informal	85	21.3
	Pupil or student	69	17.3
	Unemployed	137	34.3
Matrimonial status	Single	84	21.0
	Couple	316	79.0
Religion	Christian	367	91.8
	Muslim	31	7.8
	Others	2	0.5
Parity	Primipara	145	36.3
	Multipara	255	63.8
Distance from home to the vaccination unit (Km)	< 5	199	49.8
	≥ 5	201	50.3
Satisfaction with the vaccination unit	Yes	333	83.3
	No	67	16.8
Father’s level of education	Illiterate	2	0.5
	Primary	19	4.8
	Secondary	166	41.5
	Higher	213	53.3
Father’s profession	Private	93	23.3
	Public servant	116	29.0
	Informal	155	38.8
	Pupil or student	25	6.3
	Unemployed	11	2.8

### Immunization completeness

Of the 400 infants, immunization was complete in 96.3% of them. Amongst the infants who had completed their vaccination, 75.0% were born at the YGOPH, 90.0% of them started their vaccinations there and 87.0% regularly received their vaccines there. The immunization coverage for BCG, DTP_3_, Polio_3_ and measles were 99.8%, 93.3%, 93.3% and 100% respectively.

Vaccine coverage for each antigen is presented in Table 3, and the rates are greater than 90% for each antigen. The measles and yellow fever vaccines had the highest coverage of 100%.

**Table 3:** Vaccination coverage and timely administration per antigen

Vaccines	Vaccines received n^a^(%)	Vaccines received timely n^c^(%)
BCG + Polio 0 (N^b^ = 400)	399 (99.8)	332 (83.2)
Vaccines at 6 weeks (N^b^ = 366)	360 (98.4)	340 (94.4)
Vaccines at 10 weeks (N^b^ = 269)	261 (97.0)	249 (95.4)
Vaccines at 14 weeks (N^b^ = 208)	194 (93.3)	182 (93.8)

^a^number of children who received the vaccine

^b^total number of children at the age to receive the vaccine

^c^number of children who received the vaccine on time

Vaccines scheduled at 6 weeks = DTP-HepB-Hib_1_, Pneumo13_1_, Rota_1_, Polio _1_; Vaccines scheduled at 10 weeks = DTP-HepB-Hib_2_, Pneumo13_2_, Rota_2_, Polio_2_; Vaccines scheduled at 14 weeks = DTP-HepB-Hib_3_, Pneumo13_3_, Polio_3_

### Immunization timeliness

We noted that 73.3% of the children were fully vaccinated. The antigen-specific timeliness was 83.2% for BCG, 93.9% for DTP1 and 94.8% for the measles vaccine. The most delayed vaccines were the BCG, IPV and Pneumo13_3_.

### Determinants of immunization completeness

The mother’s level of education (secondary or higher level of education) and the father’s profession influenced positively the immunization completeness (Table 4). On bivariate and multivariate analysis, the same determinants: mother’s level of education and the father’s profession increased the infant’s chances of immunization completeness (Table 4).

**Table 4:** Determinants of immunization completeness

Variables	Immunization completeness		Unadjusted OR (95% CI)	Unadjusted *P* value	Adjusted OR (95% CI)	Adjusted *P* value
	Yes	No				
Mother’s age						
< 30 years	219(96.9)	7(3.1)	1.51(0.54-4.24)	0.43		
≥ 30 years	166(95.4)	8(4.6)				
Mother schooled to the higher level^a^						
Yes	354(97.3)	10(2.7)	5.71(1.84-17.76)	0.007	7.0(2.16-22.68)	0.001
No	31(86.1)	5(13.9)				
Profession						
Employed	251(95.4)	12(4.6)	0.47(0.13-1.69)	0.24		
Unemployed	134(97.8)	3(2.2)				
Matrimonial status						
Single	81(96.4)	3(3.6)	1.07(0.29-3.87)	1.00		
Married	304(96.2)	12(3.8)				
Religion						
Christian	352(95.9)	15(4.1)	/	0.62		
Muslim	33(100)	0(0.0)				
Parity						
Primipara	139(96.5)	5(3.5)	1.13(0.38-3.37)	0.83		
Multipara	246(96.1)	10(3.9)				
Father schooled to the higher level^a^						
Yes	365(96.3)	14(3.7)	1.34(0.16–10.41)	0.56		
No	20(95.2)	1(4.8)				
Father’s profession						
Employed	378(96.7)	13(3.3)	8.31(1.57-43.95)	0.04	12.39(2.21-69.26)	0.004
Unemployed	7(77.8)	2(22.2)				

^a^secondary + university education levels

### Determinants of immunization timeliness

Term babies, born at the YGOPH and who were regularly vaccinated there had better chances of being correctly vaccinated. After logistic regression analysis, only term babies had the greatest chance of being correctly vaccinated at the recommended ages (Table 5).

**Table 5:** Determinants of immunization timeliness

Variables	Timeliness		Unadjusted OR (95% CI)	Unadjusted *P* value	Adjusted OR (95% CI)	Adjusted *P* value
Age						
	Yes	No				
< 30 years	165 (73)	61 (27)	0.9 (0.6–1.5)	0.9		
> 30 years	128 (76.6)	46 (26.4)				
Level of education						
None/Primary	26 (72.2)	10 (27.8)	0.9 (0.4–2)	0.9		
Secondary/Higher	267 (73.4)	97 (26.6)				
Mother’s profession						
Employed	192 (73)	71 (27)	0.9 (0.6–1.5)	0.9		
Unemployed	101 (73.7)	36 (26.3)				
Matrimonial status						
Single	65 (77.4)	19 (22.6)	1.3 (0.7–2.3)	0.3		
Couple	228 (72.2)	88 (27.8)				
Religion						
Christian	239 (73.3)	98 (26.7)	1 (0.5–2.3)	0.9		
Muslim	24 (72.7)	9 (27.3)				
Parity						
Primipara	105 (72.9)	39 (27.1)	0.9 (0.6–1.5)	0.9		
Multipara	188 (73.4)	68 (26.6)				
Distance from home to vaccination unit						
< 5 Km	149 (74.9)	50 (25.1)	1.2 (0.8–1.8)	0.5		
> 5 Km	144 (71.6)	57 (28.4)				
Father’s profession						
Employed	287 (73.4)	104 (26.6)	1.4 (0.3–5.6)	0.7		
Unemployed	6 (66.7)	3 (33.3)				
Place of birth						
YGOPH	221 (75.9)	70 (24.1)	1.6 (1.01–2.6)	0.04	1.01 (0.9–3.3)	1
Others	72 (66.1)	37 (33.9)				
Gestation age						
Term	286 (81)	67 (19)	24.4 (10.5–56.8)	**<** 0.001	19.3 (8.1–46.1)	< 0.001
Premature	7 (14.9)	40 (85.1)				
Place vaccination started						
YGOPH	261 (74.8)	88 (25.2)	1.8 (0.9–3.3)	0.07		
Others	32 (62.7)	19 (37.3)				
Usual place of vaccination						
YGOPH	252 (75.2)	83 (24.8)	1.8 (1.01–3.1)	0.04	2.1 (0.9–4.6)	0.08
Others	41 (63.1)	24 (36.9)				

No factor related to the mother or father had a statistically significant relationship with immunization timeliness.

## Discussion

An immunization completeness rate of 96.3% was noted. In South Africa, Fadnes et al. [7] in 2011 had a rate of 94%, similar to ours. In Turkey, Torun et al. [8] had a rate of 84.5%, Bofarraj et al. [9] recorded a completeness rate of 81%. Other studies had rates which were much lower than ours: Ba Pouth et al. in 2012 in Cameroon(64.3%) [5], Barreto et al. [10] in Brazil (47%), Chidiebere et al. [11] in Nigeria (30.6%), and 24.3% for Lakew et al. in Ethiopia [12]. These differences could be explained by the fact that these studies were done in communities and on age ranges different from ours. They worked on infants aged 12 to 23 months while we worked on infants aged 0 to 11 months.

The immunization coverage for BCG, DTP_3_, OPV_3_ and the measles vaccine [6] were 99.5%, 97.18%, 97.18% and 97.91% respectively. Similar figures were noted by Hu et al. in China, 90.16%, 91.63%, 92.70% respectively for DTP_3_, OPV_3_ and the measles vaccine. In Turkey, Ozcirpici et al. [13] noted lower rates, 76.7%, 62%, 62% and 62.7% for BCG, DTP_3_, OPV_3_ and measles vaccine respectively.This could be explained by the fact that they worked on larger samples. Lower rates were equally noted by Mohamud et al. [14], in Ethopia with an observed completeness rate for BCG, DTP_3_, OPV_3_ and measles vaccine of 41.8%, 41.1%, 41.1% and 24.9% respectively. This difference could be due to the fact that they worked in rural areas.

The father’s profession had a statistically significant relationship with immunization completeness. This relationship persisted after multivariate analysis. Although the vaccines of the EPI are free, there are indirect costs such as transport fees to vaccination sites. If the father is working, these indirect costs could easily be covered; as in the African context, the father is directly responsible for the needs and health of the entire family [15].

For the mother, only the level of education significantly influenced immunization completeness. This association was also found by Gidado et al. in Nigeria [16], Ozcirpici et al. in Turkey [13], Mohamud et al. in Ethiopia [14] and Hu et al. en China [6]. In Yaounde the level of scholarization is 94.3%, and ranks highest amongst all the regions of the country [17]. A litterate woman will better understand messages on vaccination during educational talks, and this increases her awareness of the importance of vaccination. In China, Hu et al. [6] found a significant relationship between the mother’s age, her profession and immunization completeness. In Nigeria, the mothers’s knowledge on vaccination, prenatal care, and information on vaccination, had a positive influence on immunization completeness [16], whereas only the mother’s age was a significant factor, in Ethiopia [14].

No statistically significant association was found between any of the infant’s variables with immunization completeness. However, the place of birth influenced immunization completeness in some studies [6, 14]. It is likely that when a child is born in a hospital, the mother is counseled on maternity and on the care of her baby, and especially on the vaccination schedule.

We observed in our study that 73.3% of the infants were correctly vaccinated. Rates of 88%, 56% and 50%, have been noted respectively in South Africa [7], New Zealand [18], and in the United States [19]. These differences could be explained by the differences in the study sites, sample sizes and study design used. Infants not immunized at the recommended immunization ages have reduced immunity, conducive for development of diseases.

The antigen-specific timeliness was 83.2% for BCG, 93.9% for DTP_1_ and 94.8% for the measles vaccine. Similar figures have been noted by some authors: BCG (99%), DTP_1_ (87%) and measles vaccine (85%) [7]; while others had lower figures, 44.59%, 45.38% and 59.25% respectively for BCG, DTP_1_ and measles vaccine [6].

Children born at term, at the YGOPH, and who were regularly receiving their vaccines there, were more likely to be well vaccinated at the recommended ages. Premature neonates often have to wait untill they are medically stable before starting vaccinations, and this could explain the delay in starting vaccination at the recommended postnatal ages. Besides, children born in the YGOPH and who are regularly vaccinated there, receive more counselling than the others. In China, Hu et al. noted that timeliness of vaccination for specific vaccines was associated with the mother’s age, maternal education level, immigration status, siblings, birth place and distance from the house to the immunization clinic [6]. In South Africa, Fadnes et al. found, the level of education of the mother and the socio-economic status of the parents [7], to be determinants of immunization timeliness.

The fact that the study was done in a single site, which was the vaccination unit of a referral hospital, and in an urban setting in which most mothers are well educated constitutes major limitations of this study. The results might not neccessarily reflect the vaccination status of the entire Yaounde community or Cameroon at large.

## Conclusion

This study shows that immunization completeness was quite high but the number of children correctly vaccinated was relatively low. We suggest that more sensitization campaigns be done so as to enlighten parents on the importance of vaccination and on the importance of vaccinating children at the recommended ages.

## Additional file

Additional file 1: Data entry form. (DOCX 21 kb)

## Abbreviations

AOR: Adjusted Odds Ratio
BCG: Bacille de Calmette et Guérin
DTP: Diphteria tetanus pertusis
EPI: Expanded program of immunization
HepB: Hepatitis B
Hib: Hemophilus influenzae b
IPV: Inactivated polio vaccine
OPV: Oral polio vaccine
PCV 13: Pneumococcal conjugated vaccine 13
Polio: Poliomyelitis
WHO: World Health Organization
YGOPH: Yaounde Gynaeco-Obstetric and Pediatric Hospital

## References

[1] Muhsen K, Abed El-Hai R, Amit-Aharon A, Nehama H, Gondia M, Davidovitch N (2012). Risk factors of underutilization of childhood immunizations in ultraorthodox Jewish communities in Israel despite high access to health care services. Vaccine.

[2] Bos E, Batson A. Using immunization coverage rates for monitoring health sector performance: measurement and interpretation issues. 2000 [cited 2016 Jan 18]; Available from: http://agris.fao.org/agris-search/search.do?recordID=US2014600832.

[3] WHO | Together we can close the immunization gap. [Internet].[cited 2016 Jan 13]. Available from: http://www.who.int/mediacentre/commentaries/vaccine-preventable-diseases/en/.

[4] Lernout T, Theeten H, Hens N, Braeckman T, Roelants M, Hoppenbrouwers K (2014). Timeliness of infant vaccination and factors related with delay in Flanders, Belgium. Vaccine.

[5] Ba Pouth SFB, Kazambu D, Delissaint D, Kobela M (2014). Immunization coverage and factors associated with drop-out in children 12 to 23 months in Djoungolo-Cameroon Health District in 2012. Pan Afr Med J.

[6] Hu Y, Chen Y, Guo J, Tang X, Shen L (2014). Completeness and timeliness of vaccination and determinants for low and late uptake among young children in eastern China. Hum Vaccin Immunother.

[7] Fadnes LT, Jackson D, Engebretsen IM, Zembe W, Sanders D, Sommerfelt H (2011). Vaccination coverage and timeliness in three south African areas: a prospective study. BMC Public Health.

[8] Torun SD, Bakırcı N (2006). Vaccination coverage and reasons for non-vaccination in a district of Istanbul. BMC Public Health.

[9] Bofarraj AMM (2011). Knowledge, attitude and practices of mothers regarding immunization of infants and preschoo l children at al-Beida City, Libya 2008. J. Pediatr Allergy Immunol.

[10] Barreto TV, Rodrigues LC (1992). Factors influencing childhood immunisation in an urban area of Brazil. J Epidemiol Community Health.

[11] Chidiebere ODI, Uchenna E, Kenechi OS (2014). Maternal sociodemographic factors that influence full child immunisation uptake in Nigeria. SAJCH.

[12] Lakew Y, Bekele A, Biadgilign S (2015). Factors influencing full immunization coverage among 12–23 months of age children in Ethiopia: evidence from the national demographic and health survey in 2011. BMC Public Health.

[13] Ozcirpici B, Sahinoz S, Ozgur S, Bozkurt AI, Sahinoz T, Ceylan A, et al. Vaccination coverage in the South-East Anatolian Project (SEAP) region and factors influencing low coverage. Public Health. 2006 Feb;120(2):145–54. 10.1016/j.puhe.2005.04.008 16260009

[14] Mohamud AN, Feleke A, Worku W, Kifle M, Sharma HR. Immunization coverage of 12-23 months old children and associated factors in Jigjiga District, Somali National Regional State, Ethiopia. BMC Public Health. 2014;14:865. 10.1186/1471-2458-14-865 PMC4158082 25146502

[15] Sarah A, Kerry D. The effects of father involvement: An updated reseach summary of the evidence. Center for families, work and well being, University of Guelph, May 2007. [Internet]. [cited 2016 Jan 13]. Available from: http://www.fira.ca/cms/documents/29/Effects_of_Father_Involvement.pdf.

[16] Gidado S, Nguku P, Biya O, Waziri NE, Mohammed A, Nsubuga P (2014). Determinants of routine immunization coverage in Bungudu, Zamfara state, northern Nigeria, may 2010. Pan Afr Med J..

[17] Institut National de la Statistique (INS) et ICF. International. 2012. Enquête Démographique et de Santé et à Indicateurs Multiples du Cameroun 2011. Calverton, Maryland, USA : INS et ICF International.

[18] Grant CC, Turner NM, York DG, Goodyear-Smith F, Petousis-Harris HA. Factors associated with immunisation coverage and timeliness in New Zealand. Br J Gen Pract. 2010 Mar 1;60(572):e113–20. 10.3399/bjgp10X483535 PMC2828859 20202354

[19] Griffin MR, Daugherty J, Reed GW, Standaert SM, Hutchins SS, Hutcheson RH, et al. Immunization coverage among infants enrolled in the Tennessee Medicaid program. Arch Pediatr Adolesc Med. 1995 May;149(5):559–64. 10.1001/archpedi.1995.02170180089017 7735413

[20] Normes et standards du programme elargi de vaccination du Cameroun [Internet]. [cited 2016 Dec 28]. Available from: http://docplayer.fr/7296942-Normes-et-standards-du-programme-elargi-de-vaccination-ducameroun.html.

