# Supplementary material for: Vaccination of infants aged 0 to 11 months at the Yaounde Gynaeco-obstetric and pediatric hospital in Cameroon: how complete and how timely?
Source: BMC Pediatr. 2017 Dec 19;17:206. doi: 10.1186/s12887-017-0954-1 (PMC5735527; doi:10.1186/s12887-017-0954-1)
Supplement: Additional file 1: — Data entry form. (DOCX 21 kb) [file 12887_2017_954_MOESM1_ESM.docx]

**Additional file 1 Data entry form**

**Title of the project :** Determinants of vaccination completeness status in children from 0 to 11 months at the Yaounde Gynaeco-Obstetric and Pediatric Hospital.

| **SECTION 1 : Socio- demographic characteristics** | | | |
| --- | --- | --- | --- |
| **N°** | **Questions** | **Proposition** | **Answers** |
| **Q101** | Age (weeks) |  |  |
| **Q102** | Sex : | 1. Male 2. Female | / ___ / |
| **Q103** | Place of birth | 1. Health unit 2. Home  3. Elsewhere  ……. | /___ / |
| **Q104** | If  in a health unit, precise…… | 1. YGOPH 2. Others | /___ / |
| **Q105** | Gestational age | 1. Born at term 2. Premature | /___ / |
| **Q106** |  |  |  |
| **SECTION 2 : Vaccination past history** | | | |
| **Q201** | Is the Vaccination up to date ? | 1. Yes 2. No | /___ / |
| **Q202** | Place of vaccination : | 1. YGOPH  2. Else where | /___ / |
| **Q203** | Usual vaccination site | 1. YGOPH  2. Else where | /___ / |
| **Q204** | Does the child has a vaccination card ? | 1. Yes 2. No | /___ / |
| **Q205** | Have the child started vaccination ? | 1. Yes 2. No | /___ / |
| **Q206** | Date of vaccination  :  -BCG :  -DPT-Hep B-Hib 1 :  -PCV13 1:  -Rotarix 1:  -Polio 1:  -DPT-Hep B-Hib 2 :  -PCV13 2:  -Rotarix 2:  -Polio 2:  -DTCoq-HepB-Hib 3 :  -PCV13 3:  -Polio 3:  - IPV : :  -Measles vaccine :  -Yellow fever  : | 1. At birth 2. Others  1. 6 weeks 2. Others  1. 6 weeks 2. Others  1. 6 weeks 2. Others  1. 6 weeks 2. Others  1. 10 weeks 2. Others  1. 10 weeks 2. Others  1. 10 weeks 2. Others  1. 10 weeks 2. Others  1. 14 weeks 2. Others  1. 14 weeks 2. Others  1. 14 weeks 2. Others  1. 14 weeks 2. Others  1. 38 weeks 2. Others  1. 38 weeks 2. Others | /___ /  /___ /  /___ /  /___ /  /___ /  /___ /  /___ /  /___ /  /___ /  /___ /  /___ /  /___ /  /___ /  /___ /  /___ / |

| **SECTION 3 : Factors influencing the child Vaccination status** | | | |
| --- | --- | --- | --- |
| **Q301**  **Q302** | **Father of the child**  :  Level of education :  Occupation : | 1. None 2. Primary  3. Secondary   4. Higher  1. Private 2. Public  3. Informal 4. Farmer  5. Farmer 6. Student 7.Unemployed 8.Retired | /___ /  /___ / |
| **Q303**  **Q304**  **Q305**  **Q06**  **Q307**  **Q308**  **Q309**  **Q310**  **Q311** | **Mother of the child :**  Age :    Level of education :  Occupation  Marital status  :  Religion :    Parity :  Region of origin  Distance between home and place of vaccination  Are you satisfied with the vaccination services ? | 1. < 30 years 2. > 30 years  1. None 2. Primary  3. Secondary   4. Higher  1. Private 2. Public  3. Informal 4. Farmer 5. Student 6. Unemployed  7. Retired  1. Single 2. Living as a couple  1. Christian 2. Muslim  3. Others ……………………………  1. Primiparous 2. Multiparous  1. Adamaoua 2. Centre  3. Far North 4. East  5. Littoral 6. North  7. North West 8. West  9. South 10. South West  11.If foreigner, Precise ……………….  1. < 5km 2. >5km  1. Yes 2.None | /___ /  /___ /  /___ /  /___ /  /___ /  /___ /  /___ /  /___ /  /___ / |
